# Evaluating Patient Empowerment in Association With eHealth Technology: Scoping Review

**DOI:** 10.2196/jmir.7809

**Published:** 2017-09-29

**Authors:** Tracie Risling, Juan Martinez, Jeremy Young, Nancy Thorp-Froslie

**Affiliations:** ^1^ College of Nursing University of Saskatchewan Saskatoon, SK Canada

**Keywords:** review, eHealth, patient engagement, patient empowerment, patient activation, measure

## Abstract

**Background:**

The prioritization of sustainable patient-centered care in contemporary health care has resulted in an increased focus on patient empowerment, which in turn is considered to facilitate patient independence, self-management, and self-efficacy. However, a definitional consensus of empowerment remains elusive, impeding efforts to translate the conceptual ideals of empowerment into a measurable entity associated with changes in health care behavior or outcomes. The rapid integration of technology in health care serves to add another layer of complexity in the measurability and operationalization of empowerment and helps to create a specific context in which this conceptual entity should be further examined.

**Objective:**

The primary objective of this scoping review was to explore the concept of patient empowerment within the electronic health (eHealth) context. A further focus on the association or measurement of this concept in conjunction with tethered patient portal use was also employed.

**Methods:**

In this scoping review, a six-step framework was used to guide the search and paper selection process. The review was initiated with two broad research questions, which are as follows: (1) What is the relationship between empowerment and the use of eHealth technologies from a patient perspective? (2) How is patient empowerment (and/or engagement or activation) influenced by accessing personal health information through a tethered patient portal? Multiple databases were employed in a comprehensive search strategy, and papers were primarily evaluated and selected for inclusion by 2 review authors, and a third author was consulted to resolve any issues in reaching consensus.

**Results:**

From an initial count of 1387 publications, this review returned nine systematic or literature review papers and 19 empirical studies that pertained to patient empowerment (and/or engagement and activation) in relation to the use of tethered patient portals providing access to electronic health records (EHRs). Of the 19 empirical publications, only four were found to have used specific patient empowerment measures with significant variety in their identified conceptual elements.

**Conclusions:**

There is a persistent lack of conceptual clarity in patient empowerment research, and this has extended to study within the eHealth context. The interchangeable use or conflation of terms such as patient empowerment, engagement, and activation, has further complicated the advancement of distinct conceptual measures. To more strongly align changes in patient empowerment with supportive eHealth solutions, the challenges of achieving a consensus on how best to operationalize and measure patient empowerment must be met.

## Introduction

In addition to complex technological evolution and advancements, health care systems are undergoing a significant paradigmatic shift in response to the demand for care transformations that deliver on the long-standing promise of patient-centered care. The move from a decidedly more paternalist system, dominated by the views and preferences of health care practitioners, to one in which patient voice has arisen as a priority, has resulted in an increased exploration of patient empowerment [1,2]. Conceptually appealing in numerous health care applications and explorations, patient empowerment is emerging as a focal point in health care research and reform [2]. Empowerment is considered to facilitate patient control through self-management and shared decision making, as well as promote equitable and collaborative approaches to health care and improved cost-effectiveness of care delivery [3]. However, significant challenges remain for those wanting to translate the conceptual ideal of patient empowerment into measurable changes in health care behaviors or outcomes.

One of the most persistent issues in the consistent operationalization of patient empowerment is a lack of a clear definition of this complex concept [1,4,5]. The multitude of applications of the term in the literature has established empowerment as a process, often of a transformative nature; a representation, or manifestation of purported key elements such as self-management and freedom of choice; an aspect or result of particular interventions themselves where often these results are measured through improved patient outcomes or reported self-management [1]. Whereas this conceptual manipulation of patient empowerment has supported a diverse array of associated study, it has been less helpful in the establishment of a concrete and comprehensive singular measure of patient empowerment.

In the pursuit of patient-centered care, this achievement of definitional consensus, a necessity to facilitate the consistent operationalization and subsequent measurement of patient empowerment, has so far remained elusive [4,5]. In addition to the broad use of the term itself, patient empowerment has also been used interchangeably with the terms patient engagement, patient enablement, patient activation, and even patient-centeredness, though numerous reports support the distinct use and application of each of these key conceptual entities [5-7]. The role of patient empowerment has been explored in specific care contexts [8], with particular chronic diagnoses such as diabetes [9,10] and cardiac conditions [11], and for patient populations spanning the full range from pediatric to geriatric. The diverse application of this popular concept has also extended into the electronic health (eHealth) literature [12-15], which, while beginning to explore important considerations regarding the influence of technology on empowerment, has also been challenged by this persistent conceptual conflation.

Discussion on the use of technology to advance patient empowerment [12,13,16,17] has taken into consideration how the concept may need to be reimagined within the eHealth context [15,18]. In addition, particular technologies such as patient portals have become a focal point in this research, with connections made between portal use, patient empowerment, engagement, and/or activation and ultimately, improved personal health outcomes [19-22]. This early work is a promising beginning in the exploration of eHealth and patient empowerment. However, the challenges of achieving a unified conceptual view of empowerment and perhaps more importantly, a single comprehensive empirical tool to evaluate empowerment in association with eHealth inventions remains. It is not sufficient to promote the empowering effects of new technologies without an accompanying evaluation of the actual influence of the intervention in this area. To advance this work, researchers need reliable measures of patient empowerment suitable for application in the eHealth context.

The initial primary focus of this scoping review was patient empowerment within the eHealth context, with a particular examination of the concept in association with patient portal use. Previously noted challenges regarding the interchangeable use of the terms patient empowerment, patient engagement, and patient activation were discovered in the early stages of the review process, and ultimately, resulted in an expansion of the search parameters. Owing to the lack of definitional consensus and consistent application of these concepts, additional search terms related to patient activation and engagement were incorporated to provide a more complete assessment of the current state of empirical patient empowerment measure in association with patient portal use. This paper includes the review findings and accompanying analysis summarized as follows: (1) characterizations of reported effects of portal use on patient empowerment, (2) identification of the range of patient empowerment, engagement, and activation measures reported in association with portal use, and (3) enumeration of differences in patient empowerment definition and measure.

## Methods

This study was conducted based on the guidelines outlined by Levac et al [23] in their update to the work of Arksey and O’Malley in 2005. There are six steps to the review process in this framework, which are as follows: (1) identifying the research question, (2) identifying the relevant studies, (3) study selection, (4) charting the data, (5) collating, summarizing, and reporting results, and (6) consultation (optional) [23]. With respect to research questions, the recommendation in this framework is that scoping reviews employ both a broad and more focused question to provide a direction for the initial identification and eventual selection of relevant studies [23]. The broad directive in this review was as follows: (1) *What is the relationship between empowerment and the use of eHealth technologies from a patient perspective?* This question supported the primary focus of the review on patient empowerment within eHealth. The second research question provided additional parameters for the search and selection of publication by focusing the review on a particular eHealth solution. This question was also expanded to include additional terms, shortly after the review began, as has been detailed. The second question is as follows: (2) *How is patient empowerment (and/or engagement or activation) influenced by accessing personal health information through a tethered patient portal?* Tethered patient portals typically provide patients with access to information contained in their electronic health record (EHR), as opposed to personal health records (PHRs) which may not. Together, these questions directed the subsequent search and selection of relevant eHealth publications presented in these review findings.

**Figure 1:**
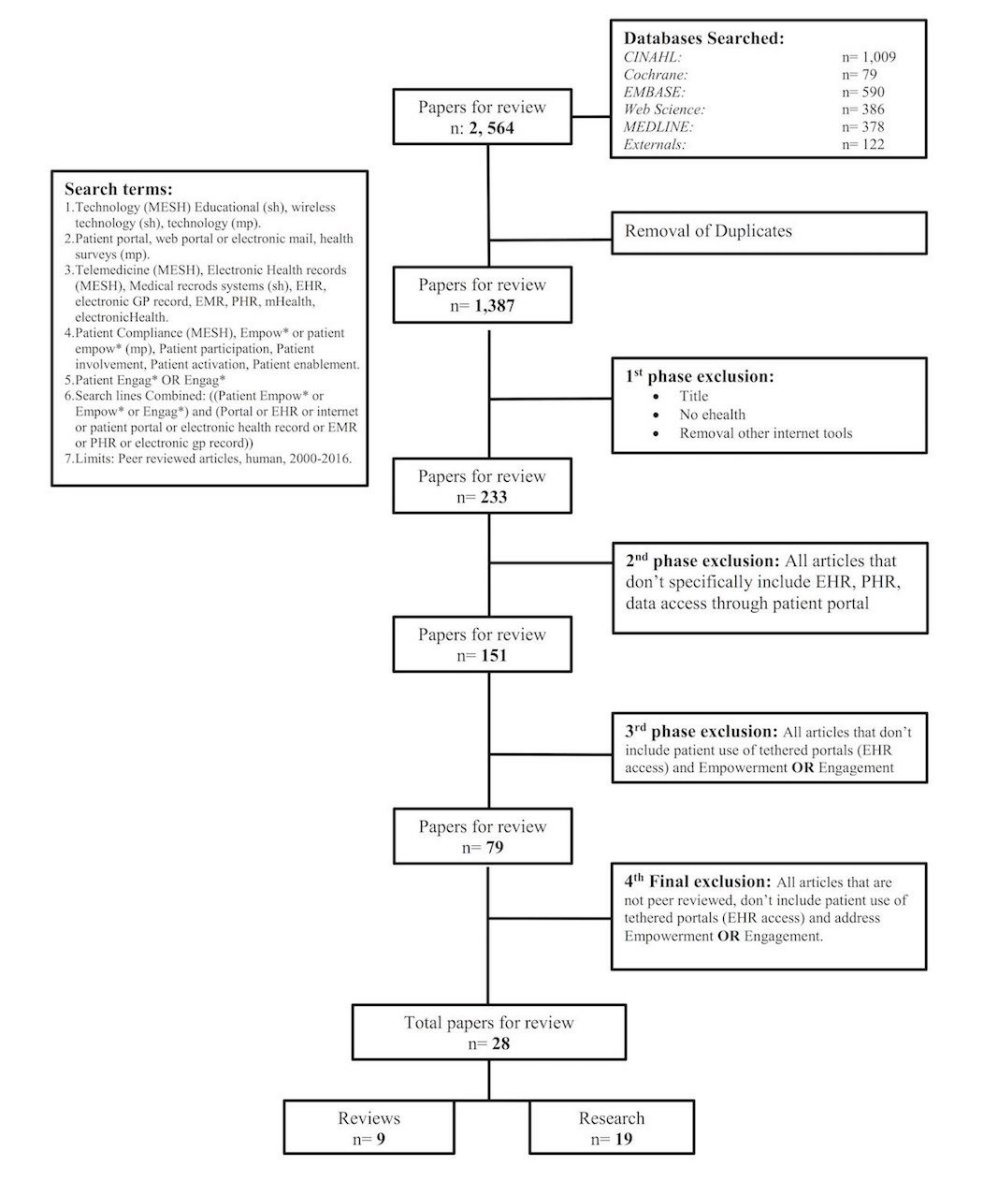
Scoping review strategy and results.

### Search Strategy

A preliminary search strategy for each selected database (Cumulative Index to Nursing and Allied Health Literature, The Cochrane Library, EMBASE, MEDLINE [Ovid], and Web of Science) was used to determine the Medical Subject Headings (MeSH) and keywords. The portal-related search terms were separated into three categories: empowerment, engagement / activation, and technology. MeSH Headings and text words were removed from the search strategy where no database results were returned. A full overview of this process is included in the detailed search strategy depicted in Figure 1. The search was restricted to English language publications between 2006 and 2016. An additional manual search of abstracts was performed to complete the review and support the inclusion of relevant publication that was not returned by the search strategy primarily because of indexing error. This work addressed the second step in the review process, identifying relevant studies, and produced a large initial publication count (n=1387) for review.

### Study Selection

In the third step of the review process, inclusion and exclusion criteria were used on the basis of the specifics of the stated research questions. These criteria can evolve, as was the case in this review, as the researchers became more familiar with the subject material during the course of the ongoing review of the publications [23]. Following the removal of duplicates obtained in the initial search, this review progressed through four phases of paper exclusion, as was detailed in the search strategy in Figure 1. The first and second authors did the majority of the exclusion, with the consultation of a third party, as needed, when consensus on exclusion could not be reached. A title review was done as a part of the first exclusion phase to remove papers that did not have a specific eHealth focus. This aided in the management of a large number of papers returned by the use of technology as a search term and resulted in a sample size (n=233) for further examination. In the second phase, an abstract review assisted the authors in removing papers that did not include studies on EHRs, PHRs, or patient portals (n=151). The third phase of the exclusion focused on identifying tethered portal publication that also included the concept of patient empowerment or patient engagement or activation (n=79). Finally, in a fourth exclusion, the authors reviewed full-text documents and ensured that each included study reported on tethered patient portals that provided patients access to their EHR and addressed patient empowerment and/or patient engagement or activation.

The final count of papers for the literature review was settled at 28, including nine literature or systematic review publications and 19 empirical study publications. The review publication was retained because of challenges in trying to delineate a clear distinction between patient empowerment, engagement, activation, and other terms, considering that it could prove to be valuable for the discussion of empirical findings.

### Data Extraction

The empirical studies were screened for data, and key points were extracted and summarized in the Multimedia Appendices 1 and Table 1 that follow. In addition to collating characteristics such as sample size, country of origin, research approach and design (Multimedia Appendix 1), a specific examination on reported tools and measures was also completed (Multimedia Appendix 2). During the synthesis process, the review of reported tools and measures led to a further enumeration of measures of patient activation, engagement, or empowerment (Table 1). This process addressed the fourth step in the scoping review framework and the concluding steps (collating, summarizing, and reporting the results, and consultation) as has been represented in the discussion of the review results.

**Table 1:** Summary of tools and concepts specifically used to measure patient empowerment, engagement, and activation.

Concept	Authors	Method or tool: concepts measured
Activation	Ancker et al [35]	Patient Activation Measure (PAM): patient knowledge, skill, confidence for self-care
	Crouch et al [28]	PAM
	O’Leary et al [36]	PAM
	Riippa et al [34]	PAM
Engagement	Toscos et al [24]	PAM
	Shi et al [37]	PAM
	Gee et al [25]	Qualitative: self-management
	Pillemer et al [40]	Qualitative: self-control, knowledge
	Rief et al [41]	Qualitative: knowledge of patient role, self-efficacy, initiative, and commitment to care
	Shade et al [38]	Outcome measure: use of health care services
	Henry et al [39]	Outcome measure: care gap closures
Empowerment	Crouch et al [28]	Healthcare Empowerment Inventory, based on Health Care Empowerment Model: engagement, informed, collaboration, commitment to treatment and tolerance of uncertainties of outcomes [32]
	van der Vaart et al [30]	Different scales for each component: patient satisfaction with care, trusting physician-patient relationship, self-efficacy in provider-patient communication, perception of illness and personal control, medication adherence
	Tuil et al [29]	Different scales for each component: self-efficacy, knowledge about treatment, involvement in decision process
	Earnest et al [31]	Composite empowerment scale: control of care, knowledge of condition, preparedness, reassurance, understanding of provider instructions, trust, ability to find mistakes

## Results

### Characterizing Effects of Portal Use on Patient Empowerment and Associated Concepts

The scoping review resulted in the extraction of nine systematic or literature reviews and 19 empirical studies. Multimedia Appendix 1 summarizes key characteristics of the 19 empirical studies. There is a wide range of literature available, characterizing the effect of patient portal utilization on patient empowerment, and/or patient engagement and activation. The progress of research publications in this area has been on an increase from 16 to the 19 publications in this area, since 2014. There is a range of international publications on this topic that is available with the majority of the studies originating in the United States.

### Identifying a Range of Conceptual Measures

In general, this review comprised a range of measures applied to the evaluation of patient empowerment, engagement, and activation among portal users. These measures were often accompanied by other assessments pertaining to specific health outcomes, medication adherence, patient and provider attitudes, or patient satisfaction. In addition, several qualitative explorations reported on patient experiences, perceived barriers associated with portal use, and perceived sense of empowerment [24-27].

### Enumeration of Differences in Conceptual Operationalization and Measures

Patient empowerment, engagement, and activation emerged as the primary conceptual entities in this review. There was considerable discussion on the barriers, experiences, characteristics of users, and health outcomes associated with the use of patient portals, however, a more elaborate exploration of each of these parameters is beyond the scope of this paper. Table 1 provides an inventory of the review papers, per concept, including the details of the measures used as reported in the publications.

The analysis of the empirical publication in this review revealed that patient empowerment, even when highlighted as a primary focus in a publication or study design, was not always specifically measured as a distinct concept in the resultant research. As summarized in Table 1, of the 19 publications reviewed, only four reported the use of focused patient empowerment measures [28-31]. When used, explicit measures of empowerment were conducted with specific composite empowerment scales [28,30], or independent measurements of interrelated components deemed to be related to empowerment [29,31]. Key components defined in relation to empowerment were distinct and varied depending on the study publication [28-31]. Crouch et al [28] utilized a previously established 8-item Healthcare Empowerment Inventory [32], designed to specifically measure empowerment through reports of being engaged, informed, collaborative, committed to treatment, and tolerant of uncertainty to outcomes or trajectory, a noteworthy contribution to this area of study.

The use and measure of patient activation was the most conceptually focused finding in this review. The widespread uptake of the Patient Activation Measure (PAM) by Hibbard Stockard [33] as a standard in this area has provided some conceptual stability for patient activation. The use of this measure as an extension to measure patient empowerment and engagement may be complicating the achievement of similar conceptual clarity for these entities. Several studies utilized the PAM in their evaluation of the potentially empowering or engaging effects of portals on patient activation [28,34-36]. In this grouping of PAM-based studies, two focused solely on activation and portal use [34,35], one on how activation and knowledge can influence engagement [36], and finally, a single study that used a separate empowerment measure in addition to the PAM [28]. This work demonstrates a focused pursuit of activation study, and in one case, acknowledges a distinction between activation and empowerment through the use of separate measures [28]. However, the issue remains that in many publications the words empowering or patient empowerment are employed to introduce or provide context for the research, with little or no conceptual follow-up or clarification found in the study results. The reported conceptualization of patient engagement was also found to be similarly inconsistent, with two studies utilizing the PAM as a validated proxy measure of engagement [24,37]. Other studies defined engagement in relation to the patients’ utilization of health care services [38], self-management of care gap closures [39], and perceived feelings of self-control and management over their health [25,40]. However, within these publications, there was a noted absence in discussing and clarifying the conceptual uniqueness of patient empowerment, engagement, and/or activation.

## Discussion

### Principal Findings

The results of this scoping review have been summarized as follows: (1) characterizations of reported effects of portal use on patient empowerment; (2) identification of the range of patient empowerment, engagement, and activation measures reported in association with portal use; and (3) enumeration of differences in the patient empowerment definition and measure. Further reflection on the conceptual complexity uncovered in this review, including findings from recent systematic and literature reviews have been detailed here.

This work has revealed the effects of tethered patient portal utilization on patient empowerment, engagement, and/or activation as somewhat controversial. Whereas none of the previous systematic reviews retained in this scoping study directly focused on the effect of eHealth technologies on patient empowerment [16,42-49], eHealth interventions, in general, have been hypothesized to contribute to patient empowerment by increasing self-efficacy and providing tools for self-management [12,29]. Overall, in early research and review, utilization of portals has most commonly been associated with small changes in patient empowerment or activation [28-31,35]. However, the use of portals was found to result in improved self-reported levels of engagement or activation related to self-management [40,41] and enhanced knowledge [25]. Portal use was also positively associated with better health outcomes in various study populations [10,24,28,37]. For example, diabetic patients with access to an electronic patient portal demonstrated improved glycemic control [10]. The diverse nature of these types of measures, and their potential relationship with any accompanying conceptual evaluation, adds to the challenge of attempting to isolate or demonstrate significant change in concepts such as patient empowerment.

The true influence of patient portals on empowerment seems to be obscured by the lack of a common vision of the concept itself. This is a significant consideration, as the conceptual clarification of empowerment is tantamount to the translation and operationalization of the concept into concrete use and practice [12]. Out of the 19 empirical studies selected from the scoping review, only four publications measured empowerment specifically, and within these, the conceptual elements of empowerment differed significantly as has been recorded in Table 1 [28-31]. Furthermore, while the *PAM* remains a standard for the measurement of patient activation [28,34-36] and engagement by proxy [24,37], specific measures of empowerment identified in the study publications varied from the use of composite empowerment scales [28,31] to the use of independent scales measuring interrelated components of empowerment [29,31] (Table 1). Hence, our review of the literature identified an overall lack of clarity and consensus surrounding the measurement and concepts related to patient empowerment in association with tethered patient portal usage.

### Limitations

The perspective presented in this scoping review is limited to patient empowerment–focused research in relation to tethered patient portal use. As a consequence, a full exploration of patient empowerment research within a broader health care context was not completed, excluding studies which may lead to differing conclusions on the current state of patient empowerment measure. This said, measures utilized in more general health care application did present within this eHealth literature. Additionally, there were challenges in the applied search strategy on account of the use of ubiquitous terms such as Internet and empowerment. Although this was done to maximize a full scope of return and to combat noted indexing errors during the search, it also resulted in the return of many results not relevant to the focused questions that then had to be eliminated in the exclusion phases. The employed search strategy did not deliver a small number of key papers that were discovered during manual searches, revealing the potential indexing challenges. Ultimately, these publications were included as hand-searched items in the review to ensure a comprehensive body of literature from which to complete the exclusion process.

### Comparison With Prior Work

This scoping review adds further evidence to prior publications that identified a lack of clarity and unification in the conceptualization of patient empowerment. There is substantial interest in achieving a more concrete operationalization of this concept [1,3,5,6]. The *Health Care Empowerment Inventory* [32] is one of the few empowerment focused measures in practice with others (not identified in this scoping review) such as the *Patient Empowerment Scale* [50], and a more recent tool, as yet unnamed, designed to evaluate patient empowerment in long-term conditions [51].The more recent work of Barr et al [5] has produced a comprehensive interdisciplinary conceptual map of empowerment, but these authors also noted that no existing single measure could adequately capture the complexity of the conceptual elements they had identified.

The findings of this review, in combination with prior studies, strengthens the position that patient empowerment is a distinct conceptual entity and should not be used interchangeably, with respect to terms such as engagement and activation [6,32]. However, numerous barriers to the development of a standardized measure of empowerment have been identified, such as the differing contexts and study populations in which the concept has been studied and diversified [12,52]; the prevalence of potentially conflicting or interrelated factors such as socioeconomic status, preexisting health conditions, Internet, or digital literacy [1]; possible issues with patient privacy and confidentiality [31]; and concerns regarding the use of technology in advancing patient empowerment, potentially resulting in a digital divide [28]. The interplay between patient empowerment, engagement, and activation must also be more clearly articulated, especially as it is related to the uptake of eHealth solutions. For example, it has been hypothesized that a high level of patient activation is required before effective portal use [35], this would, in theory, be essential for an improved sense of empowerment in relation to the solution. Barr et al [5] also indicated that advances in empowerment measure are impeded not only by an overlap of terminology in application, but by a lack of accompanying robust psychometric evaluative data.

Given the challenges in capturing and measuring the full scope of patient empowerment, it is not surprising to discover conflicting reports regarding the use of eHealth services on resultant health outcomes, as highlighted in this work, and this is consistent with the conclusions in several previous systematic eHealth reviews [42-45,47-49]. Whereas some study populations did report positive benefits from using eHealth technologies [25,27,40,41,46], it was argued that these perceived effects would not necessarily translate to overall improvements in patient empowerment or health outcomes [42,43]. This paper contributes to a growing body of eHealth research on patient empowerment. The ever-expanding presence of eHealth in the health care landscape must be factored into continuing patient empowerment study [12,13,15].

### Future Directions

The concept of patient empowerment lies within the scope of eHealth literature [12-15], with an emerging consideration that “the future of patient empowerment may lie in technological advancements and better access of patients to these technologies” [11]. This review has identified ongoing challenges regarding conflation and inconsistent conceptual application in this field and further demonstrated a current lack of consensus surrounding the operationalization and measurement of patient empowerment in particular. In addition, there are further issues with the inconsistency present in identified patient empowerment measures.

A clear association between the use of eHealth solutions, patient empowerment, and health outcomes remains elusive. Further patient-driven investigation on patient empowerment is an urgent need, particularly within certain contexts, such as eHealth intervention, where there is a paucity of literature. Until a comprehensive measure of patient empowerment is developed and thoroughly evaluated, significant challenges will remain within the eHealth context with respect to establishing patient empowerment as a means to positively influence health outcomes.

### Conclusions

The aforementioned challenges in evaluating patient empowerment have influenced the effectiveness of research on the relation of this concept with specific and measurable changes in health outcomes. Even so, recent reviews on patient empowerment reveal global interest in the advancement of research on this concept [1,5,8]. The World Health Organization (WHO) European Regional Office included empowerment and patient-centered practice as key elements in its Health 2020 report [53], a follow-up on previous WHO study on the effectiveness of empowerment to improve health [54]. The earlier WHO research identified empowerment as an essential public health strategy but also called for the ongoing refinement of measures to evaluate empowerment [54]; yet, calls for improved definition of patient empowerment and measures to comprehensively evaluate the concept remain [1,5,8], and this is clearly a need for eHealth science as well. Despite early review and research, a reliable and valid measure to evaluate patient empowerment remains elusive. “Patient empowerment strategies have been shown to positively impact health care outcomes and will likely help shape the future of medical practice” [11]; however, without adequate measure, the researchers, practitioners, and program providers will be challenged to establish the value of these interventions. This scoping review is part of a larger research project examining empowerment and patient portal use. The results of this review will be united with qualitative interview data from patient users of an EHR patient portal to produce a more comprehensive patient-directed view of empowerment to support ongoing examination of the significance of this concept in eHealth.

## Appendix

Multimedia Appendix 1 Summary of characteristics of empirical studies included in the scoping review.

Multimedia Appendix 2 Summary of concepts, tools, and measures included in empirical studies used to characterize patient outcomes related to use of patient portals.

## Abbreviations

eHealth: electronic health
EHR: electronic health record
MeSH: medical subject headings
PAM: Patient Activation Measure
PHR: personal health record
RCT: randomized controlled trial
WHO: World Health Organization
