# Supplementary material for: Evaluating Patient Empowerment in Association With eHealth Technology: Scoping Review
Source: J Med Internet Res. 2017 Sep 29;19(9):e329. doi: 10.2196/jmir.7809 (PMC5640823; doi:10.2196/jmir.7809)
Supplement: Multimedia Appendix 1 [file jmir_v19i9e329_app1.pdf]

Research design	Authors	Abridged title	Sample size, Patient/(HCP)[a]	Country	Research design	Publication
Quantitative						
	Crouch et al [28]	A pilot study to evaluate the magnitude…	20	USA	Cross-sectional observational	*PeerJ*
	Henry et al [39]	The online personal action plan: a tool…	838638	USA	Retrospective cohort	*American Journal of Preventive Medicine*
	Lau et al [10]	Impact of patient use of an online patient…	157	Canada	Retrospective observational	*Canadian Journal of Diabetes*
	Lee et al [55]	Which users should be the focus of…	7096	Korea		*Telemedicine Journal and eHealth*
	Toscos et al [24]	Impact of electronic PHR[b] on engagement...	200	USA	Prospective, quasi-experimental	*Journal of the American Medical Informatics Association*
	Riippa et al [34]	The effect of a patient portal with electronic…	137	Finland	Controlled, Before-and-after	*Journal of Medical Internet Research: JMIR*
	Ronda et al [56]	Reasons and barriers for using a patient…	1390	Holland	Quantitative	*Journal of Medical Internet Research: JMIR*
	Shi et al [37]	EHR and patient activation…	915 (384)	USA	Cross-sectional logistic regression	*International Conference on Smart Health*
	Tuil et al [29]	Empowering patients undergoing IVF[c]…	180	USA	Randomized controlled trial	*Fertility and Sterility*
	Van der Vaart et al [30]	Impact of patient accessible electronic medical records…	360	Holland	Pretest-posttest	*BMC Musculoskeletal Disorders*
	Ancker et al [35]	Patient activation and use of an electronic…	180	USA		*Journal of Medical Internet Research: JMIR*
Mixed methods						
	Earnest et al [31]	Use of a patient-accessible…	107 (7)	USA	Questionnaires, focus groups	*Journal of the American Medical Informatics Association*
	O'Leary et al [36]	The effect of tablet computers with…	202	USA	Controlled trial	*Journal of the American Medical Informatics Association*
	Pillemer et al [40]	Direct release of test results to patients…	14,441	USA	Interviews, surveys	*PLOS One*
	Shade et al [38]	HIT[d] interventions enhance care…	7354	USA	Serial cross-sectional	*Journal of the American Medical Informatics Association*
Qualitative						
	Gee et al [25]	e-Patients perceptions of using personal…	18	USA	Interviews	*Computers Informatics Nursing*
	Shah et al [26]	Accessing personal medical records…	226	UK	Cross-sectional, questionnaires	*International Journal of Medical Informatics*
	Rief et al [41]	Using HIT to foster engagement…	41	USA	Focus groups	*Journal of Health Communication*

	Woods et al [27]	Patient experiences with full electronic…	37	USA	Interviews, focus groups	*Journal of Medical Internet Research: JMIR*

[a]number of patients (number of health care providers).
[b]PHR: personal health record.
[c]IVF: in vitro fertilization.
[d]HIT: health information technology.

This is a Multimedia Appendix to a full manuscript published in the J Med Internet Res. For full copyright and citation information see http://dx.doi.org/10.2196/jmir.7809

References

10. Lau M, Campbell H, Tang T, Thompson DJS, Elliott T. Impact of patient use of an online patient portal on diabetes outcomes. Canadian Journal of Diabetes. Feb 2014;38(1):17-21. doi: 10.1016/j.jcjd.2013.10.005

24. Toscos T, Daley C, Heral L, Doshi R, Chen YC, Eckert GJ, Plant RL, Mirro MJ. Impact of electronic personal health record use on engagement and intermediate health outcomes among cardiac patients: a quasi-experimental study. Journal of the American Medical Informatics Association. 2016;23(1):119-128. doi: 10.1093/jamia/ocv164

25. Gee PM, Paterniti DA, Ward D, Miller LMS. e-Patients perceptions of using personal health records for self-management support of chronic illness. Computers Informatics Nursing. 2015;33(6):229-237. doi: 10.1097/CIN.0000000000000151

26. Shah SGS, Fitton R, Hannan A, Fisher B, Young T, Barnett J. Accessing personal medical records online: a means to what ends? International Journal of Medical Informatics. 2015;84(2):111-118. doi: 10.1016/j.ijmedinf.2014.10.005

27. Woods SS, Schwartz E, Tuepker A, Press NA, Nazi KM, Turvey CL, Nichol WP. Patient experiences with full electronic access to health records and clinical notes through the my healthevet personal health record pilot: qualitative study. Journal of Medical Internet research. 2013;15(3):e65. doi: 10.2196/jmir.2356

28. Crouch PCB, Rose CD, Johnson M, Janson SL. A pilot study to evaluate the magnitude of association of the use of electronic personal health records with patient activation and empowerment in HIV-infected veterans. PeerJ. 2015;2015(3):e852. doi: 10.7717/peerj.852

29. Tuil WS, Verhaak CM, Braat DD, de Vries RobbÈ PF, Kremer JA. Empowering patients undergoing in vitro fertilization by providing internet access to medical data. Fertility and Sterility. 2007;88(2):361-368. doi: http://dx.doi.org/10.1016/j.fertnstert.2006.11.197

30. van der Vaart R, Drossaert CHC, Taal E, Drossaers-Bakker KW, Vonkeman HE, van de Laar M. Impact of patient-accessible electronic medical records in rheumatology: use, satisfaction and effects on empowerment among patients. BMC Musculoskeletal Disorders. Mar 2014;15. doi: 10.1186/1471-2474-15-102

31. Earnest MA, Ross SE, Wittevrongel L, Moore LA, Lin C-T. Use of a patient-accessible electronic medical record in a practice for congestive heart failure: patient and physician

experiences. Journal of the American Medical Informatics Association. 2004;11(5):410-417. doi: https://doi.org/10.1197/jamia.M1479

34. Riippa I, Linna M, Rönkkö I, Kröger V. Use of an electronic patient portal among the chronically ill: an observational study. Journal of Medical Internet Research. 2014;16(12);e275. doi: 10.2196/jmir.3722

35. Ancker JS, Witteman HO, Hafeez B, Provencher T, Van de Graaf M, Wei E. The invisible work of personal health information management among people with multiple chronic conditions: qualitative interview study among patients and providers. Journal of Medical Internet Research. 2015;17(6):e137. doi: 10.2196/jmir.4381

36. O'Leary KJ, Lohman ME, Culver E, Killarney A, Smith GR, Liebovitz DM. The effect of tablet computers with a mobile patient portal application on hospitalized patients' knowledge and activation. Journal of the American Medical Informatics Association. 2016;23(1):159-165. doi: 10.1093/jamia/ocv058

37. Shi YF, Fuentes-Caceres V, McHugh M, Greene J, Verevkina N, Casalino L, Shortell S. Electronic health records and patient activation - their interactive role in medication adherence. In: Zheng X, Zeng DD, Chen H, Leischow SJ, editors. Smart Health. Lecture Notes in Computer Science. Switzerland: Springer; 2016; 954:219-230. ISBN: 978-3-319-29175-8

38. Shade SB, Steward WT, Koester KA, Chakravarty D, Myers JJ. Health information technology interventions enhance care completion, engagement in HIV care and treatment, and viral suppression among HIV-infected patients in publicly funded settings. Journal of the American Medical Informatics Association. 2015;22(e1):e104-E111. doi: 10.1136/amiajnl-2013-002623

39. Henry SL, Shen E, Ahuja A, Gould MK, Kanter MH. The online personal action plan: a tool to transform patient-enabled preventive and chronic care. American Journal of Preventive Medicine. 2016;51(1):71-77. doi: 10.1016/j.amepre.2015.11.014

40. Pillemer F, Price RA, Paone S, Martich GD, Albert S, Haidari L, Updike G, Rudin R, Mehrota A. Direct release of test results to patients increases patient engagement and utilization of care. Plos One. 2016;11(6):e0154743. doi: 10.1371/journal.pone.0154743

41. Rief JJ, Hamm ME, Zickmund SL, Nikolajski C, Lesky D, Hess R, Fischer GS, Wiemer M, Clark S, Zieth C, Roberts MS. Using health information technology to foster engagement: patients' experiences with an active patient health record. Health Communication. 2017;32(3):310-319. doi: 10.1080/10410236.2016.1138378

55. Lee G, Park JY, Shin S-Y, et al. Which users should be the focus of mobile personal health records? Analysis of user characteristics influencing usage of a tethered mobile personal health record. Telemedicine and e-Health. 2016;22(5):419-428. doi: 10.1089/tmj.2015.0137

56. Ronda MC, Dijkhorst-Oei L-T, Rutten GE. Reasons and barriers for using a patient portal: survey among patients with diabetes mellitus. Journal of Medical Internet Research. 2014;16(11):e263. doi: 10.2196/jmir.3457
