# Supplementary material for: Evaluating Patient Empowerment in Association With eHealth Technology: Scoping Review
Source: J Med Internet Res. 2017 Sep 29;19(9):e329. doi: 10.2196/jmir.7809 (PMC5640823; doi:10.2196/jmir.7809)
Supplement: Multimedia Appendix 2 [file jmir_v19i9e329_app2.pdf]

Authors	Identified concepts, measures: tool or method utilized
Ancker et al [35]	Patient activation: Patient Activation Measure (PAM)<br>Patient satisfaction<br>Frequency of portal use
Crouch et al [28]	Patient activation: PAM<br>Patient empowerment: Health Care Empowerment Inventory<br>Patient satisfaction: Consumer Assessment of Healthcare Provider and Systems Community Programs for Clinical Research<br>Patient knowledge of condition: knowledge of medications, outcome measures
Earnest et al [31]	Patient empowerment (expectations): composite empowerment scale<br>Patient attitudes: qualitative interviews<br>Physician expectations: qualitative interviews
Gee et al [25]	Self-management support: semistructured interviews<br>Productive interactions with use of personal health record: semistructured interviews
Henry et al [39]	Outcome measure: care gap closures
Lau et al [10]	Outcome measure: glycemic control
Lee et al [55]	Usage patterns: demographics
O'Leary et al [36]	Patient activation: PAM<br>Patient satisfaction<br>Patient frequency of use<br>Patient knowledge
Pillemer et al [40]	Patient perceptions of engagement<br>Patient utilization<br>Patient experiences/perceptions
Rief et al [41]	Patient experience: qualitative exploration<br>Patient engagement: qualitative exploration
Riippa et al [34]	Patient activation: PAM
Ronda et al [56]	Frequency of use, perceived barriers and opinions on usefulness: questionnaires<br>Diabetes-related distress: Problem Area in Diabetes Questionnaire<br>Self-efficacy: Diabetes Management Self-Efficacy Scale<br>Knowledge of condition: Brief Diabetes Knowledge Test
Shade et al [38]	Patient engagement: use of health services<br>Outcome measure: antiviral load
Shah et al [26]	Benefits/Challenges of electronic health record: questionnaire
Shi et al [37]	Patient engagement: PAM<br>Medication adherence
Toscos et al [24]	Patient engagement: PAM<br>Outcome measures: body mass index, low-density lipoprotein, glycated hemoglobin, blood pressure
Tuil et al [29]	Patient empowerment: different scales for each component<br>Self-efficacy: General self-efficacy scale<br>Knowledge: Visual analog scale<br>Involvement in decision making: Problem-solving decision making scale<br>Patient satisfaction: Patient satisfaction questionnaire<br>Meaning of disease: Illness cognition questionnaire<br>Social support: Inventory for social support<br>Anxiety: State-trait anxiety inventory<br>Depression: Beck depression index for primary care
van der Vaart et al [30]	Patient empowerment<br>Satisfaction with care: Quality of care through patients' eyes (QUOTE) rheumatic patients<br>Trusting physician-patient interaction: Cologne-Patient-Questionnaire scale<br>Self-efficacy in patient-provider communication: Perceived Efficacy in Patient-Physician Interactions questionnaire<br>Illness perceptions: Revised Illness Perception Questionnaire<br>Medication adherence: Morisky Medication Adherence scale<br>Perceived ease of use, clarity, usefulness and completeness of the portal<br>Patient satisfaction with portal

Woods et al [27]	Patient views and experiences: qualitative exploration

This is a Multimedia Appendix to a full manuscript published in the J Med Internet Res. For full copyright and citation information see http://dx.doi.org/10.2196/jmir.7809

References

10. Lau M, Campbell H, Tang T, Thompson DJS, Elliott T. Impact of patient use of an online patient portal on diabetes outcomes. Canadian Journal of Diabetes. Feb 2014;38(1):17-21. doi: 10.1016/j.jcjd.2013.10.005

24. Toscos T, Daley C, Heral L, Doshi R, Chen YC, Eckert GJ, Plant RL, Mirro MJ. Impact of electronic personal health record use on engagement and intermediate health outcomes among cardiac patients: a quasi-experimental study. Journal of the American Medical Informatics Association. 2016;23(1):119-128. doi: 10.1093/jamia/ocv164

25. Gee PM, Paterniti DA, Ward D, Miller LMS. e-Patients perceptions of using personal health records for self-management support of chronic illness. Computers Informatics Nursing. 2015;33(6):229-237. doi: 10.1097/CIN.0000000000000151

26. Shah SGS, Fitton R, Hannan A, Fisher B, Young T, Barnett J. Accessing personal medical records online: a means to what ends? International Journal of Medical Informatics. 2015;84(2):111-118. doi: 10.1016/j.ijmedinf.2014.10.005

27. Woods SS, Schwartz E, Tuepker A, Press NA, Nazi KM, Turvey CL, Nichol WP. Patient experiences with full electronic access to health records and clinical notes through the my healthevet personal health record pilot: qualitative study. Journal of Medical Internet research. 2013;15(3):e65. doi: 10.2196/jmir.2356

28. Crouch PCB, Rose CD, Johnson M, Janson SL. A pilot study to evaluate the magnitude of association of the use of electronic personal health records with patient activation and empowerment in HIV-infected veterans. PeerJ. 2015;2015(3):e852. doi: 10.7717/peerj.852

29. Tuil WS, Verhaak CM, Braat DD, de Vries RobbÈ PF, Kremer JA. Empowering patients undergoing in vitro fertilization by providing internet access to medical data. Fertility and Sterility. 2007;88(2):361-368. doi: http://dx.doi.org/10.1016/j.fertnstert.2006.11.197

30. van der Vaart R, Drossaert CHC, Taal E, Drossaers-Bakker KW, Vonkeman HE, van de Laar M. Impact of patient-accessible electronic medical records in rheumatology: use, satisfaction and effects on empowerment among patients. BMC Musculoskeletal Disorders. Mar 2014;15. doi: 10.1186/1471-2474-15-102

31. Earnest MA, Ross SE, Wittevrongel L, Moore LA, Lin C-T. Use of a patient-accessible electronic medical record in a practice for congestive heart failure: patient and physician experiences. Journal of the American Medical Informatics Association. 2004;11(5):410-417. doi: https://doi.org/10.1197/jamia.M1479

34. Riippa I, Linna M, Rönkkö I, Kröger V. Use of an electronic patient portal among the chronically ill: an observational study. Journal of Medical Internet Research. 2014;16(12);e275. doi: 10.2196/jmir.3722

35. Ancker JS, Witteman HO, Hafeez B, Provencher T, Van de Graaf M, Wei E. The invisible work of personal health information management among people with multiple chronic conditions: qualitative interview study among patients and providers. Journal of Medical Internet Research. 2015;17(6):e137. doi: 10.2196/jmir.4381

36. O'Leary KJ, Lohman ME, Culver E, Killarney A, Smith GR, Liebovitz DM. The effect of tablet computers with a mobile patient portal application on hospitalized patients' knowledge and activation. Journal of the American Medical Informatics Association. 2016;23(1):159-165. doi: 10.1093/jamia/ocv058

37. Shi YF, Fuentes-Caceres V, McHugh M, Greene J, Verevkina N, Casalino L, Shortell S. Electronic health records and patient activation - their interactive role in medication adherence. In: Zheng X, Zeng DD, Chen H, Leischow SJ, editors. Smart Health. Lecture Notes in Computer Science. Switzerland: Springer; 2016; 954:219-230. ISBN: 978-3-319-29175-8

38. Shade SB, Steward WT, Koester KA, Chakravarty D, Myers JJ. Health information technology interventions enhance care completion, engagement in HIV care and treatment, and viral suppression among HIV-infected patients in publicly funded settings. Journal of the American Medical Informatics Association. 2015;22(e1):e104-E111. doi: 10.1136/amiajnl-2013-002623

39. Henry SL, Shen E, Ahuja A, Gould MK, Kanter MH. The online personal action plan: a tool to transform patient-enabled preventive and chronic care. American Journal of Preventive Medicine. 2016;51(1):71-77. doi: 10.1016/j.amepre.2015.11.014

40. Pillemer F, Price RA, Paone S, Martich GD, Albert S, Haidari L, Updike G, Rudin R, Mehrota A. Direct release of test results to patients increases patient engagement and utilization of care. Plos One. 2016;11(6):e0154743. doi: 10.1371/journal.pone.0154743

41. Rief JJ, Hamm ME, Zickmund SL, Nikolajski C, Lesky D, Hess R, Fischer GS, Wiemer M, Clark S, Zieth C, Roberts MS. Using health information technology to foster engagement: patients' experiences with an active patient health record. Health Communication. 2017;32(3):310-319. doi: 10.1080/10410236.2016.1138378

55. Lee G, Park JY, Shin S-Y, et al. Which users should be the focus of mobile personal health records? Analysis of user characteristics influencing usage of a tethered mobile personal health record. Telemedicine and e-Health. 2016;22(5):419-428. doi: 10.1089/tmj.2015.0137

56. Ronda MC, Dijkhorst-Oei L-T, Rutten GE. Reasons and barriers for using a patient portal: survey among patients with diabetes mellitus. Journal of Medical Internet Research. 2014;16(11):e263. doi: 10.2196/jmir.3457
